# ‘Feelings stronger than reason’: conflicting experiences of exercise in women with anorexia nervosa

**DOI:** 10.1186/s40337-016-0100-8

**Published:** 2016-03-09

**Authors:** Liv-Jorunn Kolnes

**Affiliations:** Norwegian School of Sport Sciences, PB 4014 Ullevål Stadion, 0806 Oslo, Norway

**Keywords:** Anorexia nervosa, Compulsive exercise, Ambivalence and control, IPA

## Abstract

**Background:**

Individuals with anorexia nervosa frequently feel ambivalent about treatment and weight restoration, and drop out and relapse rates in treatment are high. Increased insight into the function of the eating disorder is considered essential for achieving long-lasting, meaningful change. However, research investigating the functions of anorexia nervosa tends to focus on the role of the disease *per se*. Distinctions are rarely made across features. In particular, the subjective experience, understanding and sense making of the engagement in compulsive exercise in individuals with anorexia nervosa has received little attention. By using a qualitative methodological approach, this paper aims to expand on prior findings by examining how patients with anorexia nervosa understand and make sense of the experience of exercise in the context of their lives and treatment programme.

**Methods:**

Semi-structured interviews were conducted with six females, four of whom were former athletes. Transcripts were subjected to interpretative phenomenological analysis (IPA).

**Results:**

Two overarching themes emerged in the analysis of the larger study of which this paper is a part; ‘paradoxical functions of exercise’ and ‘diverging experiences of exercise’. Diverging experiences of exercise is the focus of this paper. Firstly, in spite of being severely underweight and suffering from exhaustion, as well as having a clear awareness of the associated negative health effects, participants were engaged in a continuous cycle of rigorous and excessive exercise, which consumed extensive amounts of time and energy. Secondly, the results demonstrate how exercise routines negatively control and interfere with the participants’ involvement in the social world. Thirdly, the manner in which participants speak about their exercise reveals their wording to be characterized by efforts to downplay the extent of their actual immersion in exercise. Issues of control and ambivalence about treatment and recovery can be considered potential triggers for the participants’ engagement with exercise. Implicit meanings are elaborated upon and discussed in relation to existing literature.

**Conclusions:**

The material provides increased insight into the multi-layered meanings of exercise for individuals with anorexia nervosa. It also suggests alternatives to current ways of understanding and approaching exercise that may enable this issue to be addressed in a more meaningful way in therapy. Qualitative approaches can make a valuable contribution to furthering such understanding.

## Background

While anorexia nervosa is a serious illness commonly associated with high levels of medical and psychological complications [1, 2], it is also recognised that individuals with anorexia nervosa may have positive feelings about their illness [3–5]. It is therefore not surprising that such individuals frequently feel ambivalent about treatment and weight restoration, and that drop-out and relapse rates in treatment are high [6, 7]. Attempts to produce long-lasting, meaningful change during the treatment of eating disorders focus on reducing psychiatric distress, producing changes in self-concept, and providing increased insight into the function of the eating disorder for the individual [8]. However, research into the functions of anorexia nervosa tends to focus on sense making or the role of the disease *per se* (e.g., [9–11]). Distinctions are rarely made across features, such as between explicit functions of restrictive eating and intense exercise routines, all of which are considered core parts of anorexia nervosa (restrictive subtype). In particular, little attention has been paid to the subjective experience, understanding and sense making of compulsive exercise in individuals with this disorder. Accordingly, existing work may not fully capture the means by which individuals make sense of the diverse practices and features that are part of their illness, or how these relate to one another.

Compulsive exercise is associated with increased levels of disordered eating attitudes and behaviours and considered an important component in the development and maintenance of anorexia nervosa (e.g., [12, 13]), particularly the restrictive subtype [14–16]. Preoccupation with exercise in the eating disorders has long been associated with weight- and body shape concerns [17–19]. In view of the assumption that individuals with anorexia nervosa have difficulties in identifying and regulating their emotions [20–23], it is assumed that the regulation and neutralizing of negative emotions also makes an important contribution to compulsive exercise [24–28]. There are, however, concerns that compulsive exercise may become the usual method by which emotions are regulated and that this may prevent the individual from exploring alternative ways of managing difficult feelings [8, 29]. Currently, there is no agreement among researchers and clinicians regarding what constitutes excessive versus compulsive exercise in this context. The most consistent support for a term that captures adherence to strict and recurring exercise routines maintained in spite of negative consequences, appear to be for the term *compulsive exercise* [30]. Excessive exercise may also be comprehended as a continuum characterized by increased prioritizing of exercise routines over other activities in parallel with increased exercise tolerance, which may gradually become more noticeable and progress into compulsive exercise [31].

While physical activity is commonly associated with health benefits, in the context of anorexia nervosa, exercising is a complex, multi-layered dimension that needs further understanding. It has been shown that heavy exercising in individuals with anorexia nervosa is accompanied by an increased risk of medical complications [2, 18], longer length of hospitalization [19], moderate clinical results [32], and an increased prospect of relapse after recovery [7, 33]. Studies of exercise in people with anorexia nervosa have in the main used standardized measures or fixed self-report surveys. These may not entirely capture the meaning of exercise for people who exercise compulsively. With few exceptions [34–37], qualitative investigations of the experience of exercise in individuals with anorexia nervosa are sparse. In an auto ethnographic paper, Axelsen [35] describes challenging experiences connected with the shift from being an anorectic to becoming a devoted triathlete. She gives an account of how her engagement with exercise developed from walking the dog as a ‘time out’ from difficult feelings to gradually becoming more compulsive and interfering negatively with her societal life. Having received psychological and medical care for her illness, she regained some weight, although her exercising became increasingly excessive after she quit treatment. Eventually, Axelsen reached a turning point and began training for a triathlon. This new training gradually helped Axelsen regain weight and reconnect with her body in what was perceived to be a more healthful manner than her previous exercising [35]. It also entailed engagement in social interactions with training peers. Successively, she identifyed her self more with being an athlete, than with being an anorectic. Given that the core beliefs and feelings that maintained the exercise routines did not seem to have been sufficiently challenged, it could, however, be suggested that she had mainly replaced one compulsion with another in the process of becoming a devoted triathlete. Yet, she maintains that the engagement in triathlon became a journey into some sense of recovery, and that the new compulsion was more balanced and healthy than the former, whilst also representing a sense of belonging and a positive lifestyle [35]. Employing a thematic analytic approach, Moola et al. [36] explore experiences of physical activity in Canadian women previously hospitalized for their anorexia nervosa. Despite expressing serious health issues due to exercise routines, the women engaged in compulsive exercising motivated by weight concerns and anxiety reduction. The authors describe confusion among patients and staff about what is considered physical activity and what represents eating disorder psychopathology and how this gave rise to challenging situations [36]. Furthermore, the women’s engagement in any physical activity was frequently considered to compromise treatment goals, and attempts to deal with problematic exercise in hospital treatment were characterized by exercise restriction [36]. In a narrative inquiry study, Young et al. [34] examine the role of exercise across the lifespan in a mostly Australian sample of women with anorexia nervosa. This study illustrates the transformation of exercise from being balanced and enjoyable during childhood into compulsive and rule-driven exercising during their illness [34]. Whilst providing emotion regulation and a sense of being in control in the participants, concomitant to previous research, exercise contributed to prolonged progress and relapse and was considered a prominent feature in sustaining the illness [34]. The findings of a qualitative report focusing on the meaning women with anorexia nervosa make of their experience with exercise are complemental to these studies. In addition to taking part in high levels of exercising to escape and regulate feelings, the results illuminate how feelings deriving from past and present challenging life experiences resonate as bodily sensations, and how participants turn to exercise to ease off embodied distress stemming from such feelings [37]. This study further demonstrate the significant meaning of exercise to the women’s sense of self and identity, and how their sense of self entangle with an anorectic and an athletic identity, a complementary identification giving increased connotation to the reluctance and ambivalence towards change apparent in some of the participants [37].^1^

Given the challenges and confusions connected with the understanding and management of exercise during treatment [36] and the assumption that compulsive exercise is a major emotion-regulation strategy that contributes to maintaining the illness [29], an understanding of how individuals with anorexia nervosa make sense of their engagement in such exercise is now needed. Indeed, it is imperative to challenge the view that exercise is a more acceptable behaviour than disturbed eating or dieting in this context [38]. By using a qualitative methodological approach, the current paper aims to expand the aforementioned findings by examining how individuals with anorexia nervosa restrictive subtype understand and make sense of the experience of exercise in the context of their lives and treatment programme.

## Methods

To address the issue central to this study, interpretative phenomenological analysis (IPA) has been employed. IPA is concerned with understanding the person-in-context, and exploring individuals’ relatedness to the world. IPA is a phenomenological and hermeneutical research approach [39, 40]. Phenomenological philosophy is valuable as it offers a rich source of beliefs about how to examine and understand lived experience, which is unique to the person’s embodied and situated relationship to the world [40, 41]. IPA is inspired by *phenomenology* as it is concerned with trying to understand the individuals’s personal perception or account of an experience, as opposed to attempting to produce an objective record of the experience itself [40]. IPA connects with *hermeneutics* in that phenomenological examination is an interpretative process. In the examination of how a phenomenon appears, the analyst offers an interpretative account of what it means for the participant to have such concerns [42]. IPA also links up with an *ideographic* commitment, situating participants in their particular contexts, exploring their personal perspectives, starting with a detailed examination of each case before moving to more general claims [40]. As the aim is to reveal something of the experience of each given person, IPA studies usually involve a small number of participants. The rationale of sampling in IPA is based on producing a clearly situated, circumscribed and transparent account of what has been found within contexts, and within homogenous populations [42]. It is not assumed that there will be a place to stop where one can claim that this is enough data, or that ‘saturation’ has been achieved. IPA is concerned with keeping the numbers low, in order to look for rich detail, similarity and difference, shade and nuance, such that ‘saturation’ is reached in terms of what has been taken from the individual case or these cases. IPA has increasingly been used among health and social care professionals as it allows for an in-depth exploration and a nuanced account of individuals with a health condition, at the same time as it pays attention to explicit content and implied meanings.

It may be noted that a unique part of the broader study of which this paper is a part, involves a body examination frequently applied in Norwegian psychomotor physiotherapy with the aim of providing insight about bodily tension and constriction in the participants. Due to its separate character and focus on how clients’ functional impairments may guide exercise and physical therapy interventions in a beneficial way, the results of the body assessment and a discussion of relevant implications for treatment will be detailed in a separate paper.

### Participants

Six women who were undergoing intensive in- or outpatient treatment for anorexia nervosa participated in this study. All of these women were attending residential or day treatment at eating disorder services in Norway. Intensive treatment is commonly recommended for patients with anorexia nervosa who have a low BMI, and weight restoration is often the primary goal. The inpatient treatment programmes impose non-negotiable weight gain expectations and restricted engagement in exercise (usually restricted to walking 15 – 30 min, two to three times per day). To achieve depth and richness of data, it was essential that the sample of interviewees was homogenous and shared particular criteria related to the research question [43]. Participants selected were over the age of 18, they had a clinical diagnosis of anorexia nervosa restricting subtype and were considered compulsive exercisers. The diagnosis was provided by their consultant psychiatrist or psychologist using ICD 10 [44]. For inclusion in this study, compulsive exercise was defined as ‘moderate to vigorous exercise for more than six hours per week for a minimum of 1 month before hospitalisation’ [45]. This definition does not capture qualitative aspects of exercise, yet it is utilized in relevant literature [46]. It takes into consideration that exercise for more than six hours weekly may have harmful effects (e.g., overuse injuries, decreased bone density, stress fractures) for individuals with anorexia nervosa who are severely underweight [2, 47]. Four participants were involved in competitive sports in their teens, prior to the onset of the illness. The sample only includes women, as there were no men admitted to the units during the recruitment phase meeting the inclusion criteria. The participants’ ages, duration of illness and length of treatment are listed in Appendix 1. Their body mass index (BMI) was between 15.2 and 19.2 at the time of the interview, while lowest BMI had been between 12.6 and 14.5. The participants were asked to take part in the study by their physician or psychotherapist. One woman, still fulfilling the inclusion criteria, but who recently had resigned from the service, was asked to take part by the author. To protect participants’ anonymity and safeguard confidentiality, names and identifying features were altered. The study was given ethical approval by the Regional Ethics Committee of Southern Norway and administrative consent was granted by the hospital units involved. Participants were given written information regarding the research and were informed of the possibility of withdrawing from the interview at any time. Informed consent was gathered from all women.

### The interview schedule

The interview schedule consisted of open-ended questions. Participants were initially encouraged to recount the history and status of their eating disorder and experiences with exercise, including their interest in sport prior to the onset of anorexia nervosa. Next, they were asked to elaborate on their thoughts and feelings related to exercising, the functions and meanings it has for them, and how their training may influence other aspects of their lives (e.g., Can you describe your exercise and activities during a day and week? What are your thoughts/feelings about exercise, and about why you exercise? Does your exercise influence other aspects of your life? If so, in what way?). The interview procedure was flexible in order to allow interesting points emerging during the interview to be followed up. For instance, in the course of the interview, prompting by asking particular questions (e.g., How long are your walks? Do you do any strength training? If so, what do you do specifically? What about activity when in your room? What do you do when on leave?) was used in order to obtain balanced responses about the scope of the participants’ experience with exercise. The author conducted the interviews during second half of 2013 in a private room at the units or at the author’s workplace. The interviews lasted for 50–90 min, they were audiotaped and subsequently transcribed verbatim by the author.

### Data analysis

The analysis closely adhered to the four-stage process described by Smith et al. [40]. In the first step, the author read the primary transcript a number of times in order to become familiar with the data. Any significant or interesting initial comments were recorded during this reading. Secondly, initial notes were transformed into emerging themes while paying attention not to overstate relations between the participants’ own words and the researchers’ interpretations. During the third stage, the emergent themes were examined for patterns and connections, and clustered according to conceptual similarities. These clusters were subsequently labelled according to the conceptual nature of the themes in each cluster. Next, a table of themes was produced and sub-themes were nested with supporting quotes from the participant. Quotes were coded (e.g., A: Agnes/3: page number/C: quote 3 on page) to safeguard the possibility of revisiting the transcript to check the context of the extract. Transcripts for the next five participants were then read and subjected to the same analytic process. When considering individual cases during data analysis in an IPA study, we are inevitably influenced by what has been found in previous cases. To allow new themes to emerge, it is crucial to maintain the rigour of systematically following the steps outlined by IPA for each case [40]. Thus, individual cases were considered on their own terms, allowing new themes to emerge within each case. After conducting the analysis for each case, cross-case patterns were established and documented in a table of themes for the group. This information was transformed into a narrative account supported by verbatim extracts from the participants. An experienced IPA researcher reviewed and audited the analytic journey to ensure firm grounding in the transcripts. Two overarching themes emerged during the analysis, ‘paradoxical functions of exercise’ and ‘diverging experiences of exercise’, of which the second is the focus of this present paper.

### Issues of quality and validity

To demonstrate scientific rigour and trustworthiness, the criteria outlined by Yardley [48] for assessing validity and quality in qualitative psychology are considered useful for ensuring high quality of research with IPA [40]. These criteria include sensitivity to context, commitment and rigour, coherence and transparency, and impact and importance. Smith [49] has made suggestions to guide IPA research output, by recommending that sufficient quotations from the data are used to illustrate the points made and to show density for each theme (e.g., for N = 1–3, quotations are needed from every participant per theme; for N = 4–8, quotations are needed for at least three participants per theme). The present study adheres to Yardley and Smith’s guidelines concerning scientific rigour.

### Reflexivity

The author of this paper has a doctoral degree in sport sciences. She is a physiotherapist specialist in Norwegian psychomotor physiotherapy (NPMP^2^), and has a number of years of experience of working with individuals with anorexia nervosa. Reflexivity involves the need for the researcher to recognise that she always brings her own horizons of experience and fore-conceptions to the encounter and that phenomena are continuously interpreted in the light of these. While presumptions may hinder the process of allowing the new (e.g., person, text) to speak in its own voice, they might also represent a way in to the new. The author recognises that her past experiences and understandings inevitably colour her perceptions. Nevertheless, these may also be helpful in the interpretation process, by allowing her to empathise and understand the participants’ voices and to help her explore in depth the meaning of significant events and processes [40].

## Results

Two superordinate themes were identified in the analysis of the larger study of which this paper is a part: ‘*Paradoxical functions of exercise’* and *‘diverging experiences of exercise’*. To ensure richness of the phenomenological description and the exploratory interpretative account of the participants concerns within each overarching theme, the two themes are examined in two separate papers. This present article reports on *diverging experiences of exercise*, due to its rich examination of participants’ challenging relationship with exercise. The analysis of *paradoxical functions of exercise* reporting on exercise as a means of regulating feelings and easing off embodied emotional distress, as well as the experience and meaning of exercise for the participants’ self and identity, are published in a separate paper [37]. Subthemes in all participants are illustrated in Appendix 2.

### Diverging experiences of exercise

Key features of this superordinate theme are experiences of exercise as a rigorous and comprehensive entity limiting the participants’ social and relational worlds. Accounts of how the participants experience and relate to compulsive exercise and what it is like to be engaged in such exercise are examined first. Secondly, the manner in which exercise is prioritized over and limits involvement in the social world is explored. Thirdly, the paradoxical wording that characterizes the participants’ descriptions of their engagement in exercise is presented, along with their tendency to minimize and redefine their engagement with physical activity.

#### ‘I feel like I am being compelled to exercise’: Ambivalence and control

This theme illustrates participants’ diverging experiences with exercise and how compulsions, rules, routines and conflicting feelings accompany this engagement. The analysis is introduced by extracts from Agnes and Hedda illustrating some of the main features of the participants’ accounts^3^:

Agnes: When it first started, I was very thin. (..) In the end, I found myself eating and training, eating and training. It became a constant vicious circle (..). Every time I was full, I exercised or threw up. (..) I have been doing several things on top of the training sessions, such as working out compulsively in the midst of other activities and doing push-ups wherever possible. This has increased more and more, (..) there were around 200 before breakfast (..) in between taking a shower, preparing breakfast, and then in the afternoon and before going to bed. (..) I had to do push-ups while visiting friends, or at the university. (..) Ultimately, it was like being on autopilot, as I constantly had to do more than the day before and exercise longer or harder or run further, even though I was exhausted and desperate since I was short of energy. Once I fainted on the spinning bike. Still, I could not take a step back and see that something was wrong. I could not take a break and breathe and look at what was actually going on, and why I needed this. I just remained with this obsession.

Hedda: If I go to the gym (..) I cannot only strength train (..), I must run first, even if I have run in the morning. I also cycle, to and from work, which is probably 20 or 30 km (..). I do not run less than 10 km, preferably 20 km on weekdays. If I have the chance, I run closer to 30 km at weekends. (..) It has to be a whole number of kilometres, it cannot be 9.7 km or 10.5 it has to be 10 or 11. (..) I also used to have several rules in the gym, but since it was so demanding to have so many schemes about which equipment to use and for how long and stuff, I cut it out. (..) It was like 45 min on the treadmill, 30 min on the step machine and 10 min on the rowing machine, then back to the treadmill for 20 min, a little weight-training (..), and three times this and that. I was very stressed out by it. (..) I felt that it was only exercise characterized by hatred. It was just tiring. I did not enjoy it at all.

In these extracts, Agnes and Hedda describe very powerfully how exercise has become increasingly compulsive, controlling and structured around particular rules and routines. They engage in a perpetuating cycle of exercise and food restraint, consuming extensive amounts of time and energy in their lives. Hedda experiences part of her training at a fitness centre as exhaustive and aversive, but managed to cut this part of her training out. Agnes describes her engagement in training as an automatic entity that is beyond her control and persists in exercising despite being physically exhausted or nearly collapsing. This experience gives rise to a sense of lacking control and feelings of powerlessness. Agnes clearly expresses an understanding of her training routines as out of place. She also reflects on why she needs to train and why she cannot let go of it, apparently unable to connect with the underlying thoughts and feelings triggering the obsession to exercise. In contextualizing this account within Agnes’ narrative as a whole, it becomes evident that Agnes struggles with loneliness and feelings of not being good enough when comparing herself to family and friends.

Those participants who were athletes before the onset of the eating disorder (e.g., Hedwig and Rebecca) were engaged in healthy exercise when they first entered the sport and training arena in their childhood or early teens, though their training successively escalated into a more intense endeavour. Here, Hedwig and Rebecca illuminate how they have pushed themselves to extreme fatigue during training and competitions:

Hedwig: I remember I took part in a couple of long-distance competitions and I was nervous about how it would work out. People thought I was crazy, but it went really well… I only used to eat one meal per day, so it is difficult to understand how I managed… I do not really get it, since I had not eaten. I must have been exercising in a kind of adrenaline intoxication. (..) At times, when I was training at the gym, I could get so exhausted that I nearly fainted, sort of. Because when I do not eat anything during the day and exercise hard, I gets totally worn-out. (..) More or less, I have been doing the same type of training, no matter how exhausted I have been.

Rebecca: I used to exercise a lot when I was an athlete. It was like several hundred hours per year when in high school. Though, after a while I was not allowed to train. (..) I remember being constantly unwell. They thought it was a virus, kind of. But I had that drive to exercise and continued to train, so I did not get any better… I lost more and more weight that year.

These extracts further emphasize the intensity of the drive or compulsion to exercise. Bodily signs of fatigue and exhaustion are ignored, as the women persist in training and competing regardless of being physically ill and lacking energy. Despite a clear awareness of exercise being at odds with their current health condition and nutritional status, they exercise intensively.

In the following extract, Nora describes similar experiences whilst comparing aspects of her training with ‘torture’ and with a ‘vicious circle’ of exhaustion:

Nora: It is simply so tiring and boring (..). They are all the same movements and over such a long time, it is almost like torture. Yet, I must exercise. (..) I feel that if I have not trained one day, then I cannot eat. But then I must eat. I would like to refrain from exercise and get out of this cycle, but I do not know if I can. It is hopeless. (..) It is such a vicious circle wherein I need to exercise to be able to eat and rest, and then it is gone. Then along comes the next cycle and the next cycle. There is no end to the tormenting of myself. (..) I try to be more moderate or not exercise at all, I do not know which is the easiest. Every day I decide this has to end, but when I wake up it is the same roundabout again. (..) It really surprises me how it is possible to see that training is not the right thing for me now, yet, I still choose to exercise. When I think rationally, my body should rest, that would be best for me… But then again I have other feelings that are stronger than reason, which force me to exercise. Indeed, it is compulsory.

Here, Nora feels caught up in a never-ending ‘roundabout’ of exercising and eating that bears resemblance to feelings of being tortured or tormented. Indeed, an internal battle is taking place within Nora, in which she struggles with conflicting feelings and thoughts about her body needing rest and the simultaneous obligation to exercise. At the same time, she recognises that the ‘outcome’ of exercising is short-lived (i.e., ‘then it is gone’). Clearly, ambivalence seems to be of relevance to Nora’s engagement with exercise. She too recognises that her exercise regime influences her health negatively, nevertheless, she feels forced to exercise, as the urge to exercise is ‘stronger than reason’. Moreover, and analogous to Agnes’ experience in the first extract, Nora appears to experience powerlessness as she feels unable to moderate or stop the training. This feeling of lacking control over exercise signifies her belief that giving up exercising is impossible in her current situation. Importantly, she seems unable to explain why she needs to train or why she has such intense feelings that feed the compulsion to train excessively. In the next extract, Hilde exemplifies how exercise generates feelings of powerlessness, while at the same time also provides feelings of being in control:

Hilde: I actually never sit down. If I watch TV, I either walk on a step machine, or cycle on a spinning cycle, or I walk back and forth while trying to see what is going on. I cannot sit still. Before I attended treatment, I only sat down during meal times, or else I felt I did not deserve to sit still. I was incredibly restless, so it was difficult to relax. (..) No matter where I was heading, I walked or I cycled, also back and forth from work. In addition, I did a lot of walking while working. (..) I feel like I am being compelled to exercise, as if someone has decided that now you must exercise or everything will be chaos. Since I can be really short-tempered, I feel I will be awful to be with if I do not exercise. (..) It is like a remote control (..). The main challenge right now is to be in treatment five days per week… I feel that the threat is enormous, because there are five days each week that I cannot decide… Thus, I exercise a lot during leave to compensate for the time at the unit.

Hilde’s engagement in exercise is extreme. She feels she is not worthy of sitting still and is continually active in one way or another. In addition to replacing public transportation with physical activity (e.g., walking or cycling), she has a work situation that allows for extensive walking during the day. Her need to be physically active seems to have a regulatory function in terms of escaping and controlling difficult feelings, or as a means of expressing problematic feelings. It may be hypothesized that the role of exercise in consolidating herself and escaping difficult feelings relates to problematic experiences in her past. During the interview, she conveyed having been sexually assaulted in her early teens, and that she only recently had begun acknowledging and articulating these experiences in therapy.^4^

Based on the phenomenological perspective that the body offers us the means to interact with and understand the world [41], it may be suggested that Hilde’s personal story and the associated unsolved feelings are imprinted in her body, her earlier life experiences are embodied [50]. Accordingly, the use of extensive exercise in particular when on leave from the hospital, to relieve and escape bodily distress and embodied feelings, gives her a sense of disruption and a feeling of control. Thus, her exercising appears to have multi-layered meanings, as she not only trains to compensate for exercise restrictions and meal plans when in hospital, but also to consolidate herself in order to endure the awakening of embodied feelings taking place in current psychotherapy. In view of this, the prospect of losing the means (here: exercise) by which she controls some of her feelings appears overwhelming. It is interesting how she uses the metaphor ‘remote control’ to describe her drivenness towards exercise. References to feelings of being externally controlled suggest that her obligation to exercise seems beyond her control. It may be assumed that the battle around lacking control versus taking control relates to her body and to a process where the body is brought back from being an object from which she has separated herself in the years following the assaults (objective body) into a body that currently engages with her lived world (subjective body) (see e.g. [51] for details on this distinction).

In this last quote, Agnes expands on the experience of feeling controlled by exercise and at the same time retaining control through exercising when on leave:

Agnes: It has been very difficult to follow the treatment plan and not being allowed to go out. (..) Thus, I have been over-active when home on leave, as I need to compensate for all those hours that I am not entitled to exercise at the unit. Though this is not something I want to carry on with. It has been extremely difficult to resist the need to do small errands, walk back and forth in the halls, and do anything that represents some sort of physical activity. I can see the control this has had over me, such as when on leave, I have been running up and down the stairs, or I have written applications for leave in order to meet friends in town and instead walked around the city for hours. (..) Even though I say yes to treatment, on the other hand, I insist on having the control that I feel I have.

Although treatment guidelines for anorexia nervosa commonly require individuals to refrain from compulsive exercising and to commit themselves to a meal plan, Agnes retains control by exercising heavily during leave. While the engaging in such exercise regimes prevents her from exploring alternative ways of dealing with problematic feelings, the prospect of exercising on leave seems to facilitate her commitment to the treatment process. In explicitly disclosing that while accepting treatment, she insists on retaining control, Agnes illustrates very powerfully how control and ambivalence are key to the participants’ engagement in exercise. Such ambivalence may indicate that the individuals are perhaps not ready for recovery.

#### ‘I should rather have trained’: Limiting the societal and relational world

This theme examines how exercise negatively interferes with the participants’ relationships with the social world in a variety of ways. In the following extract, Agnes illustrates how adhering to strict exercise schemas impedes and obstructs her engagement in the societal world:

Agnes: It has controlled me a lot, so that I have been unable to accept invitations. I have scheduled when to exercise, when to eat and go to bed, it has turned into routines that I have not been able to change. Therefore, it has affected me such that I cannot take part in many societal things. I have spent a lot of time by myself, since training is more important. (..) Also, I have made many weird and stupid excuses to family and friends about why I could not take part in events. As a result, I have been lying a lot… to those around me… Today I find it problematic to say that I have lied, or that they will realise that I have been lying, or why I have lied. Thus, it has been problematic to let anyone get close to me.

Agnes communicates very powerfully how daily routines around training and meals limit involvement in the societal world and how this produces a feeling of being socially isolated from family and peers. Moreover, Agnes illuminates challenging experiences connected with being in close relationships. Since the masking of intense training has been going on for years, it is extremely difficult for her to engage in close relations with family and friends, as this cover up invokes feelings of embarrassment and shame. Accordingly, in order to avoid disclosing the various practices that form a part of her anorexia, she chooses to avoid relating to people emotionally. Yet, this withdrawal from social situations can result in a loss of social connectedness and of the prospect of acquiring close friends, leading to further social isolation and distress. The following extracts from Hedwig and Hedda further demonstrate how, when tailoring their daily lives to fit their training regimes, exercise is preferred to taking part in the social and relational sphere:

Hedwig: It destroys everything. (..) Because, if I come home from work and a friend all of a sudden calls and asks if we can go out and eat sushi, if I did that, it would definitely have clashed with a planned training session, and that would be a disaster. I have very strict routines around exercise. The training is always more important, it is utterly rigid.

Hedda: If something social comes up (..), then I must think through the day in detail… If I say yes to be social, and if I cannot squeeze in at least a five-kilometre run or something like that, I must do some planning and make sure I can do some additional running the day after. That way, I manage to let go of a training session. But as a rule, if it is a weekday, I usually just say that I have to work and there will not be any socialising.

Hedwig is deeply concerned about the prospect of social events getting in the way of her training activities, which are considered more crucial. The phrasing emphasizes how her training habits are categorically fixed and not up for negotiation. Hedda seems to experience slightly more flexibility regarding her exercise routines. Even though she commonly chooses training instead of engaging in social events, she sometimes manages to postpone some of her exercise activities if something social comes up.

In the next extract, Hilde further describes dilemmas associated with excessive training, food restraint and taking part in the social world:

Hilde: If I have the opportunity to train, I easily choose not to attend weddings and such things. (..) Yet, it is not only about exercise, it is also about the fact that, basically, food is always included in social settings. When I was truly ill, I was so withdrawn that I shut myself in and avoided everything that included food. (..) Even though spending time with friends can help me refrain from exercise, this may also cause an irritation to build up, because during the whole visit I would only be concerned about that I should rather have been training. (..) I have tried to spend time with friends, but then I only train later. Thus, it does not really work as a long-term distraction.

Here, Hilde describes difficulties connected to engaging in situations that include food, as these make it more challenging to take part in the social world. Moreover, while spending time with friends and family has the potential to be a positive force in her life, helping to reduce the obligation to exercise, spending time with others may also trigger the compulsion to exercise even more. As such, the societal appears ambiguous, as it merely becomes a short interruption that causes further negative feelings to build up. This experience emphasizes the intensity of the compulsion to exercise and the difficulties that may evolve from social participation. Compared to Agnes and Hilde, Hedda seems able to engage somewhat more with the relational world, as she lives with her long-term partner whom she describes as being understanding about her illness, and who takes on an active role in the recovery process. However, living in a close relationship is also challenging in terms of potential disagreements about exercise:

Hedda: If I am going out for a run, he tries to negotiate a bit. So before I run, I have to eat or drink some of those energy products, or something like that. When we are at the gym he comes over and tells me that ‘now you have run enough, now you should do some strength training’… Before, I could push him away, in a way. Now, I know he is right, so there is no point in getting into an argument with him. (..) Still, if he says ‘no, I do not think you should exercise today’, I get really angry. In the end, he gives in. He does not manage to resist my anger, sort of.

While exercise is to some extent a subject open to negotiation, it is also clearly a matter producing tension and conflict in Hedda’s relationship with her partner. A clear sense of ambivalence is taking place, such as when Hedda first admits that her partner is right that she should reduce her training, but in the very next sentence insists on having her own way if they disagree. While her partner attempts to support her in the recovery process, she is not interested in him having too much impact upon her level of exercise. This need to have full control over the amount and intensity of training may reflect a typical position of ambivalence in individuals going through the treatment and recovery process of anorexia nervosa. As described by a number of participants in this study, they choose treatment, whilst at the same time insisting on retaining the sense of control provided by their exercise routines.

The participants’ accounts illuminate how exercise in general is prioritized over taking part in the social world and over involvement in close relationships, and how social relations produce feelings of ambivalence. While spending time with friends may provide some distraction, being social may also increase the intensity of negative feelings, leading to further exercising. It may be that these experiences not only relate to avoidance of internal discomfort and difficult feelings, but also to avoidance of ‘taking up space’ and of connecting with others, since this involves emotional commitment.

#### ‘I only went for long walks’: Minimizing and rephrasing

This theme illustrates the participants’ paradoxical wording with respect to their engagement in exercise. In speaking about their various exercise routines, their speech is habitually flavoured with phrases that minimize the scale and intensity of the training activity, or which imply that their activity is not ‘real’ exercise. Hedwig vividly illustrates this point:

Hedwig: Earlier (..), I did not exercise as such, I only went for long walks, maybe two to three hours per day. (..) When I am at the unit, I just do some stretching. I try to stretch every day, but it is not exercising. I only stretch the body, stretch the arm like that, once on each side. I also stretch in order to do the splits, since it is important for me to continue to do them (..). I do not know if that counts as exercise. (..) Then I do sit-ups every night, maybe five or ten sit-ups (..) Besides, when brushing my teeth, I now and then do knee bends, say I do ten knee bends. But I do not consider this to count as exercise. At times, I do a few push-ups. It is not much really, maybe I do around sixty push-ups, other days I do not do any.

Here, Hedwig gives a detailed account of her engagement in comprehensive physical activities during her day at the unit. It is noteworthy that in spite of reporting an extensive amount of push-ups, she instantly refers to these as ‘a few push-ups’ and ‘it is not much really’, emphasizing that what she does is not regarded as real exercising. Nora and Rebecca equally talk down their training, as illustrated in the next quotes:

Nora: Since I was admitted to the hospital, I have been doing some training similar to aerobics… I lift my arms and legs, and do a few steps. (..) I only exercise for about one hour or so. Then I do some strength training for the stomach and the back. In one day, we are talking about two hours altogether. The strength training is very easy, I get no increase in pulse, but it gives me a feeling of having done something.

Rebecca: Since I am not entitled to exercise, I walk a great deal instead. I walk everywhere. In a day, maybe I walk for three hours. That is how I manage to keep my activity levels down, sort of, such that I do not exercise or run, or other such things.

While describing her activity as ‘I lift my arms and legs’, ‘I do a few steps’ and ‘the strength training is very easy’, Nora concurrently admits to undertaking two hours of aerobic and strength training each day. Rebecca conveys how despite being aware that exercise is not acceptable due to her low body weight, she still walks extensively to compensate for ‘real’ training, from which she manages to abstain when in treatment. As such, she substitutes one energetic physical activity (i.e., running) with a seemingly more moderate activity (walking for hours), proposing that such ‘moderate’ activity is not actual training. Similarly, Hilde makes a distinction between her own level of exercise and the training she thinks other individuals with anorexia are undertaking:

Hilde: Compared to those that you have to deal with, it is not much. Generally, I run between 10 or 15 km four times a week. Mostly, I run 10 km three times a week and one long distance run. (..) For the moment, I am not supposed to train at all, but I probably exercise, yes, it must be as much as three times per week.

In comparing herself to what she assumes to be the level of exercise in other study participants, Hilde assumes that she trains less than they do. Yet, while at once admitting that she should not be training when receiving treatment, she describes an activity level apparently at odds with her current treatment programme. Eventually, she recognizes that her level of activity can be considered exercising. Relatedly, Nora uses daily routines to justify parts of her activity when on leave. By referring to imaginary errands, she conceals the actual level of activity to her family:

Nora: It was really difficult. I made up excuses, for instance, if I went for a walk, I said I would go for a walk and that I would go via the grocery shop, or something like that. The truth was, however, that I was walking the whole time, and that I did not go via the shop. (..) I said this every so often to hide how much it really was.

The accounts of this theme capture the manner in which the women articulate their training, and how this may blur, minimize, conceal or redefine the real level of exercising. Once again, ambivalence is of relevance as the participants display a clear understanding of the need to be cautious about exercising due to their present health situation, but at the same time engage in prolonged exercising. Given a treatment programme obliging the individuals to refrain from exercise, the participants’ tendency to minimize their level of physical activity may be a way of expressing a seeming loyalty to the treatment plan. By talking down their engagement in exercise, they avoid disclosing their real activity levels, as well as potential discussions with staff about increased nutrition following the elevated energy expenditure. More importantly, such minimizing may prevent the theme of exercise commitment from being adressed in therapy and participants from exploring the diverse functions that exercise may have for them.

## Discussion

The purpose of this study is to explore how participants with anorexia nervosa restricting subtype experience and understand their engagement with exercise in the context of their lives. The analysis presents a range of conflicting experiences expanding on previous findings on the experience of exercise in this area. Firstly, in spite of being severely underweight and suffering from exhaustion and feelings of hopelessness, as well as having a clear awareness of the associated negative health effects, participants are engaged in a continuous cycle of rigorous and excessive exercise (e.g., aerobics, strength training, stretching, running or walking multiple hours per day) consuming extensive amounts of time and energy in their lives. They habitually replace transportation with exercise and convert work situations into physical efforts. Some participants even compare their drive towards exercise with torture and hatred. These findings are consistent with recent qualitative work showing how individuals with anorexia nervosa persist in exercising despite pain and injuries in muscles and joints and frequently describe their partaking in such exercise regimes as ‘gruelling’ and ‘demon-like’ [35, 36].

While some participants do not seem to connect with the underlying thoughts and feelings triggering the compulsion to exercise, others experience exercise as a moderator for difficult feelings and bodily distress connected with negative events of the past (e.g., sexual assaults). This resonates with former studies (e.g., [24, 25]) and is exemplified by Hilde, who exercises to self-regulate in order to endure the emotional distress arising from ongoing psychotherapy. The idea that problematic past experiences are reflected in the body adheres to phenomenological literature, proposing that we interact with and understand the world through our bodies [41]. In line with phenomenological perspectives, as well as theoretical underpinnings of Norwegian psychomotor physiotherapy [50], the material calls attention to the salient value of a person's life experiences (i.e., physical, psychological and social strain) and how these are embedded in the body and may invoke bodily tension and distress. If such feelings are continuously controlled and suppressed by rigorous exercise and restrictive eating patterns and underlying conflicts and meanings are not addressed, this may prevent the individual from engaging fully in the treatment process.

As has been shown in previous work [5, 34, 52, 53], the material demonstrates diverging experiences of being in control and losing control in individuals with anorexia nervosa. While these findings may be illustrative of the power of the obligation towards exercise, they also reflect accompanying feelings of powerlessness regarding the ability to moderate or change such activities. Though exercise appears functional in terms of emotion distraction, bolstering of self and identity, and in retaining a sense of structure and control, rigorous exercising also generates feelings of lacking control. For instance, the material illustrates the experience of an external power (i.e., ‘remote-control’) struggling against and controlling the participants more reasonable self, similar to notions of an ‘anorectic voice’ discussed in foregoing literature (e.g., [54]). Also, a number of the participants recount sensations of their exercising being imposed by an ‘autopilot’ or compulsions ‘stronger than reason’, similar to the notion of an ‘internal voice’ driving the anorexic behaviour put forward in previous work [36, 55]. Yet, while accepting treatment, the women insist on having a sense of autonomy and control, and retain such control through exercising extensively when on leave from the units. Paradoxically, exercise seems to facilitate coping with enhanced internal stress and conflicting feelings associated with gaining weight when in the recovery process. These observations indicate strong feelings of ambivalence towards altering exercise routines and foster doubts to whether the participants are ready to fully submit themselves to the requirements of the treatment programme.

Secondly, the findings demonstrate how exercise routines negatively control and interfere with the participants’ involvement in the social and relational world. In contrast to a beneficial approach to exercise, in which exercise is organized to fit into life in general, for the women in this study, life in general is organized around exercise. As found in prior studies [4, 9, 10, 35, 53], extensive engagement in exercise instead of taking part in the societal world causes difficulties in maintaining close relationships. For instance, instead of disclosing the concealed and unduly taxing exercise routines to family and friends, some participants rather choose lonesomeness. While struggling with associated feelings of shame and guilt, close relationships that might involve intimacy and emotional connection are avoided. For those remaining in close relationships, exercise may be an issue open to negotiation, whilst also producing disagreements and conflicts, as shown in Hedda’s account. Furthermore, while socialising may represent a distraction from the obligation to exercise, this interruption is merely temporary and may intensify problematic feelings still further, resulting in further exercising.

Such restricted participation in the social world is problematic for these individuals as it may produce social isolation and a loss of social and interpersonal connectedness, elements considered critical for young adults in their becoming a self and in the shaping of social support [56, 57]. A restricted social life may lead to a lack of relational skills and of confidence in making and maintaining relationships in general [10]. Limited social connectedness may further reinforce negative attributions of the self and others, resulting in a sense of aloneness that connects the participant even more strongly to the anorectic identity. Whereas involvement in the social world represents a challenge causing conflicting feelings and ambivalence in the participants, previous research has established that maintaining connections with social supports and involvement in social activities outside treatment may also have positive impacts in terms of preventing social isolation, developing interpersonal skills, and promoting recovery in individuals with an eating disorder [58–61].

Thirdly, this study illuminates the manner in which participants speak about their experience of exercise, and how their wording is characterized by a reduction and rephrasing of their actual immersion in exercise. Diverse training regimes are commonly described as not being ‘real exercise’. The verbalizing of training appears to be rather paradoxical and works to blur and conceal what is actually going on. Given the participants’ health status following enduring underweight status and the accompanying medical vulnerabilities [2], the high activity level reported by the participants clearly represents a threat to their physical health. These findings concur with a few other papers showing how language may serve to minimize the level of exercise [62, 63]. In support of such curtailing of the level of physical activity in the eating disorders is a paper examining self-reported (by the writing of a diary) versus objectively assessed (by using an accelerometer) moderate to vigorous physical activity. This study reveal a higher objectively measured than subjectively reported physical activity level in individuals with an eating disorder compared to healthy controls [62].^5^ Such reductive phrasing of the engagement in training is of clinical importance as it may prevent experiences of exercise from coming to the fore, thus limiting the opportunity to explore the meaning and function of exercise, and to investigate alternative ways of thinking and interacting with problematic feelings and the social world.

In sum, the analysis shows that conflicting feelings and emotional distress build up when taking part in a treatment process where weight gain is required and exercise strictly regulated. Thus, participants engage in a perpetuating cycle of extensive exercising which interferes with their health, social and interpersonal life, as well as the process of recovery. Accordingly, issues of control and ambivalence about treatment and recovery can be considered potential triggers for the women’s’ engagement with exercise when undergoing treatment. Such ambivalence and the associated lack of readiness to relinquish the problematic behaviour maintaining the eating disorder are considered key dimensions in the symptom presentation of individuals with anorexia nervosa [4, 5, 8, 64]. Notably, this ambivalence has been shown to persist throughout treatment in individuals with anorexia nervosa, who will gain weight in treatment [64]. Being caught up in ambivalence is, however, problematic as it may prevent the women from engaging themselves fully in the recovery process.

Concerning implications of this study for treatment, it is essential that core beliefs about self and behaviour that maintain such ambivalence need to be challenged. To offer a more meaningful treatment for those who engage in compulsive exercising, it is imperative to increase the understanding of beliefs and feelings perpetuating exercising for the particular individual in her situated context. It has been suggested that the foundation for achieving such an understanding, is a functional therapeutic relationship and an acknowledging of the significance of exercising for the individual [8]. One therapeutic approach leading to long-lasting change in a female former athlete (‘Deborah’) who exercised almost continuously in combination with restrictive dietary routines, has been illustrated by Geller [8]. Drawing on cognitive-behavioural and motivational approaches, a reformulation of maladaptive beliefs was achieved, along with a reprioritization and strengthening of values associated with relationships and personal development. As provided in Geller [8], the strengthening of patients’ self-confidence and relational skills may help them make autonomous decisions about their lives so that the need for maladaptive coping strategies, such as exercising compulsively, is reduced [8]. Thus, instead of focusing heavily on food and weight gain issues in the therapeutic programme, there is a need to target wider life-related and psychological issues which are important to the individual client and which could enable her/him to challenge the basis of her/his emotional distress [5, 59, 65, 66].

Along with increased understanding of the function and meaning of exercise for the individual client, the manner in which exercise and physical activity are comprehended and approached in treatment programmes may also need some reconsideration. Restricting activity entirely in patients with anorexia nervosa while they are in a treatment situation involving increased emotional distress may cause further difficulties, and has been shown to be counterproductive due to its potential to weaken ‘the already fragile grasp of perceived control’ ([67], p. 60) in these individuals. Restricting a problematic, but nevertheless reachable, coping strategy for managing the self and the emotions may hamper the capacity and motivation of these individuals to manage the treatment situation, particularly since their repertoire for articulating difficult feelings and thoughts is otherwise limited. The difficulty for persons with anorexia nervosa to fully anticipate the experiences they will have upon reducing exercise and gaining weight may explain some of their ambivalence towards change. The clients may possibly benefit from increased knowledge about bodily functioning, qualities of physical activities and how these relate to nutritional requirements. To increase treatment compliance, one option may be to carefully implement, in a manner complementary to cognitive-behavioural and motivational work (e.g., [8, 68]), a nutritionally supported exercise programme to accompany the process of medical stabilization. Some foregoing studies suggest that exercise interventions of moderate intensity may engender positive aspects for medically stable patients, such as increased psychological well-being, reduced anxiety and increased compliance to treatment [13, 69–71]. A tailored exercise programme combined with an opportunity to reflect on the experience before, during or after the session, has shown a reduction in obligatory attitudes towards exercise [72]. Thus, a diversity of supervised and individually tailored activities with the aim of increasing body awareness, muscular strength, bodily stability and balance could be introduced to medical stable patients. Such interventions could, however, appear more meaningful and health beneficial for the client if they were founded on bodily assessments accomplished by physiotherapists having explicit expertise regarding the examining of the body and identifying bodily impairments and resources. It could be noted that improved awareness of the body may be favourable for multiple reasons, as it may perhaps help integrating the body as one’s own, and improve emotional awareness and the ability to attune oneself to problematic feelings and the social world [73, 74].

The methodological approach and limited sample of this research may connect with both the strengths and limitations of the study. The use of a qualitative methodology, such as IPA, is particularly suitable for exploring and situating the means by which participants make sense of their experiences. In applying this approach, rich detail, nuance and difference to the examination of the experience of exercise in women with anorexia nervosa and how they make sense of these experiences from their personal and contextualized perspectives are provided. An in-depth exploration of personal experiences and meanings in this area seems suitable as it may engender detailed and renewed insight into the participant’s relatedness to and engagement with exercise. In order to produce a clearly situated and transparent account, the findings are from a small homogenous sample of participants. Yet, it is hoped that this idiographic illumination of the understanding and sensemaking of the experience of exercise in individuals with anorexia nervosa can provide increased insight into the intricacy surrounding exercise that can inform and benefit clinical and therapeutic interventions. It is assumed that the visibility of the context of the participants and the research process offer readers to estimate transferability to other similar contexts [40]. Overall, qualitative approaches, such as IPA, can make valuable contributions to furthering the understanding of participants personal concerns, and what these concerns means for the individual in a given context.

## Conclusion

In conclusion, the present study adds detail to our knowledge of exercise in women with anorexia nervosa, and to how they make sense of these in the context of their lives. It seems that, despite the functional aspects of regulating feelings and bodily distress, as well as the fuelling of the self through exercising [37], the women’s engagement in a continuing cycle of exercise are accompanied by challenging experiences affecting their health, social lives and recovery process negatively. It appear that a sense of being in control and strong feelings of ambivalence towards change are essential triggering factors regarding the women’s preoccupation with exercise. However, such immersion in exercise may keep the women trapped in their ambivalence, subsequently preventing them from exploring other ways of dealing with problematic feelings and strengthening the self. These findings illuminate the necessity of comprehending and addressing the phenomenon of exercise in patients with anorexia nervosa on multiple levels. In addition to adapting exercise interventions in accordance with the medical situation and level of weight gain (e.g., [13, 34, 72]), these interventions could be grounded on individual body assessments in order to become more health beneficial and motivating for the client. Moreover, personal beliefs and feelings that are preservative to the ambivalence triggering the problematic exercise behaviour needs to be acknowledged and investigated in more detail during treatment.

## Appendices

### Appendix 1

**Table 1:** Participants

Pseudonym	Age	Duration of eating disorder, years	Time in treatment
Agnes	26	13	1 year
Hedda	30	15	2 years
Hedwig	27	12	6 months
Hilde	39	23	2 years
Rebecca	23	6	5 years
Nora	50	4	3 years

### Appendix 2

**Table 2:** Superordinate theme: Diverging experiences of exercise

	Agnes	Hedda	Hedwig	Hilde	Rebecca	Nora
*Subtheme 1*: ‘I feel like I am being compelled to exercise’: Ambivalence and control	x	x	x	x	x	x
*Subtheme 2*: ‘I should rather have trained’: Limiting the societal and relational world	x	x	x	x	x	x
*Subtheme 3*: ‘I only went for long walks’: Minimizing and rephrasing	-	-	x	x	x	x

## Footnotes

1 The findings presented in this article is part of the larger study of which the present article is also a part.

2 Norwegian psychomotor physiotherapy (NPMP) acknowledges the body as the centre of experiences and knowledge through which we take in information and interact with the surroundings. As such, personal experiences and stories are imprinted in and reflected in our bodies, they become embodied. The method is based on the understanding that posture, respiration, muscle tension and body awareness are closely related to emotional and psychological strain [50].

3 … Illustrates a pause in speech. (..) Illustrates that words not adding meaning are omitted.

4 Hilde’s experience of exercise in terms of emotion regulation and escape has been explored in more detail in the paper dealing with the first superordinate theme [37].

5 It may be noted that only 8 out of 59 participants in the eating disorder sample had anorexia nervosa [62].
